# Urethral Mobilisation and Glanuloplasty Technique for Primary and Second-Stage Repair of Hypospadias: A Single Hospital Experience

**DOI:** 10.7759/cureus.63189

**Published:** 2024-06-26

**Authors:** Saeed Alhindi, Sanad Elrishe, Zahra Khalaf

**Affiliations:** 1 Pediatric Surgery, Salmaniya Medical Complex, Manama, BHR; 2 Department of Surgery, Royal Blackburn Teaching Hospital, Blackburn, GBR

**Keywords:** congenital, proximal hypospadias, urethral mobilisation, glanuloplasty, hypospadias repair

## Abstract

Background

Over the years, the technique used to correct hypospadias has undergone several modifications to improve outcomes and reduce complication rates. In this study, a modification has been made to the second stage of the two-stage repair of proximal hypospadias. This modification utilises urethral mobilisation and glanuloplasty, traditionally used to repair distal hypospadias, in the second stage of repair of proximal hypospadias. This study aims to assess the implications of this modification on the outcomes in addition to adding to the pre-existing literature on the outcomes of urethral mobilisation and glanuloplasty in the repair of distal hypospadias.

Methodology

A prospective study was conducted at Salmaniya Medical Complex in Bahrain between January 2016 and December 2021. All patients with either proximal or distal hypospadias who underwent a surgical repair using the urethral mobilisation and glanuloplasty technique were included. The following cases were excluded: patients with proximal hypospadias who did not undergo a first-stage repair, those with a hypoplastic urethra, and those aged 14 years or more.

Results

The mean operative time for the proximal hypospadias group was 78 minutes, while it was 62 minutes in the distal hypospadias group. Furthermore, the catheter remained in situ for a mean of three days postoperatively in the proximal hypospadias group. Overall, four of 35 patients (11.4%) experienced complications in the proximal hypospadias group. Of these, there were two (5.7%) cases of meatal stenosis, two (5.7%) cases of wound dehiscence, and no cases of diverticula or urethrocutaneous fistulas. Meanwhile, in the distal hypospadias group, one of 117 patients (0.9%) experienced a complication; the complication was meatal stenosis. there was a significant correlation between the age of patients and the complication rate (p = 0.06). The operative time was also found to be a significant factor influencing the occurrence of complications. The follow-up duration ranged between five months and 12 months. All patients had good cosmetic outcomes.

Conclusions

This study found that urethral mobilisation and glanuloplasty for the second-stage repair of proximal hypospadias resulted in lower complications than the traditional two-stage operation and a short duration of urinary catheterisation. There is a need to conduct studies with longer follow-up durations and objective measures of function to provide a better comparison between the different techniques used.

## Introduction

With an incidence rate of 1 in 300 live births, hypospadias is known to be one of the most common congenital abnormalities in male children [1,2]. It is typically characterised by the displacement of the meatal orifice, the curvature of the penis, and a hooded foreskin [3]. Although hypospadias classifications vary, they can be categorised based on the meatal opening position into distal and proximal hypospadias. Distal hypospadias includes glanular, coronal, and distal shafts while proximal hypospadias includes midshaft, proximal shaft, penoscrotal, and perinea [3]. The current recommended age for children to undergo the surgical repair of hypospadias is between six and 18 months [2].

Over the years, the technique used to correct hypospadias has undergone several modifications to provide better outcomes and reduce complication rates [4]. Some of the common techniques used to correct distal hypospadias include meatal-based techniques, tubularisation, and flap techniques. Meanwhile, the correction of proximal hypospadias is typically performed using a one- or two-stage urethroplasty, whereby the choice between the two often depends on the surgeon’s preference and experience [5,6]. The technique via which surgeons choose to correct hypospadias is usually decided intraoperatively depending on the anatomy of the child and what is found in the operation theatre [7]. According to Springer et al., besides the surgeon’s experience, their background and training can also influence the surgical approach [4]. Steven et al. found that the majority of surgeons in their study favoured the tubularised incised plate (TIP) procedure and the two-stage approach when correcting distal and proximal hypospadias, respectively [8]. Proximal hypospadias procedures have a higher rate of complications (between 15% and 90%) compared to distal hypospadias [9]. Regardless of the meatal position, distal or proximal, the most common complication of any hypospadias surgery was found to be urethrocutaneous fistula (UCF) [1].

In this study, a modification has been made to the proximal hypospadias repair following the onlay procedure. This modification utilises the urethral mobilisation and glanuloplasty following the onlay procedure in a single operation. This approach is traditionally used to repair distal hypospadias, in the onlay repair of proximal hypospadias. Whereas the traditional repair of proximal hypospadias involves the formation of a neourethra. This approach is hypothesised to reduce complications such as UCFs and stenoses which may be linked to long suture lines and inadequate support to the repaired area [10,11].

This article aims to evaluate the results of using urethral mobilisation and glanuloplasty to repair distal hypospadias cases as well as exceptional cases of proximal hypospadias with the meatus at a low position (between the tip of the glans penis and corona area) as a result of complications (e.g., cases of glanular dehiscence or distal ischemia) when indicated. We aim to assess the effectiveness of this technique and examine the associated complications.

## Materials and methods

A single-centre, prospective study was conducted between January 2016 and December 2021 after obtaining ethical approval by the responsible ethical committee (approval number: 58080421). The inclusion criteria were as follows: all patients either had onlay repair (with the meatus at the coronal area) or had distal hypospadias and underwent a surgical repair using the urethral mobilisation or glanuloplasty technique during the prespecified period. The exclusion criteria were as follows: all patients with proximal hypospadias who underwent a previous stage hypospadias repair (by onlay technique), those with a hypoplastic urethra, those aged 14 years or more, and cases of onlay which were complicated by fistula or tethering. We did not keep records of objective measures of function such as testosterone before or after the procedure.

The operative intervention used was a single operation which involved urethral mobilisation and glanuloplasty to repair distal hypospadias cases as well as exceptional cases of proximal hypospadias with post-onlay repairs with complications leading to a low location of the neomeatus (e.g., cases of glanular dehiscence or distal ischemia) when indicated. In cases where an onlay was done in the first part of the surgery, the chordee was corrected without the division of the urethral plate, and instead, a dissection of the urethral plate of the corpora approach was adopted. Following this approach, the urethral plate was preserved and other methods such as dorsal plication as well as urethral plate division were avoided. However, in the cases where the location of the meatus was not at the tip of the glans (between the tip of the glans penis and corona area) due to distal ischemia or partial glanular dehiscence, urethral mobilisation and glanuloplasty were used to advance the meatus at the tip of the glans penis. Intravenous ceftriaxone was administered at the time of induction of anaesthesia according to local protocols. At the beginning of the procedure, the gap between the native meatus and the tip of the glans was measured. The mean distance between the meatus and the tip of the penis was 5 mm. A traction 5/0 prolene suture was fixed on the glans, and then a urethral stent sized 6-10 French was inserted into the bladder according to the patients’ ages and the size of the urethral meatus. Then, a circumferential subcoronal incision was made just proximal to the hypospadias meatus, following which penile degloving was done. This was followed by the application of a tourniquet at the lowest point of the area degloved. Then, another circular incision was made around the urethral meatus and the urethra was mobilised from its bed on the glans using fine scissors. The dissection was continued proximally until an adequate length was reached (this length corresponds to the ratio between the mobilised urethra’s length and the distance between the native meatus and the tip of the glans). It is noteworthy that even though dissecting around the meatus is traditionally known to be difficult, it was successfully performed by following a meticulous and cautious approach, which was achieved using magnifying loops during the procedure. Then, the glans wings were deepened enough to create a bed for the mobilised urethra. The meatus was fixed to the glans tip by a few circumferential stitches using 7/0 polydioxanone suture materials to make the neomeatus, and the glans wings were then closed by two to three interrupted stitches using 5/0 Vicryl sutures. Subsequently, the tourniquet was released, and the urethral stent was secured using a traction suture. Finally, circumcision was done only in cases of distal hypospadias.

Following the operations, compressive dressings were applied. Furthermore, intravenous ceftriaxone was continued postoperatively while patients were admitted and oral antibiotics were used following the day-case surgeries. Patients received adequate analgesia postoperatively. The dressing and stents were removed three days postoperatively.

The data collected included demographics, operative parameters, postoperative protocols, and complication rates. The demographic data were mainly the patients’ operative ages. The operative parameters were the operative techniques, operative times, and the type of anaesthesia used. The postoperative protocols were the duration of urinary catheterisation, duration of hospitalisation, and follow-up duration.

The SPSS statistics software version 27 (IBM Corp., Armonk, NY, USA)was used for statistical analysis. Tables were used to demonstrate descriptive data including means and ranges. The complications were depicted as frequency tables with their corresponding percentages. The data were examined for statistical significance and p-values of 0.05 or less were deemed significant.

## Results

A total of 152 patients underwent urethral mobilisation and glanuloplasty either for distal hypospadias (glanular, distal penile, or post-circumcision) or proximal hypospadias after onlay repair. Of these, 35 cases were hypospadias post-onlay repairs and 117 were cases of distal hypospadias. The distal hypospadias cases included 45 patients with glanular hypospadias, 47 with distal penile hypospadias, and 25 with distal post-circumcision hypospadias. The patients who had already undergone onlay repair (with the meatus positioned at the coronal area) had a mean age of 3.05 years (range = 1.1 to 10 years), while the patients with distal hypospadias had a mean age of 1.45 years (range = 6 months to 4 years). The mean operative time for the post-onlay repair group was approximately 78 minutes, while it was approximately 62 minutes in the distal hypospadias group. Furthermore, the catheter remained in situ for a mean of three days postoperatively in the post-onlay group and 2.22 days postoperatively in the distal hypospadias group. All 35 (100%) patients who underwent onlay surgery for proximal hypospadias were hospitalised for one day only. Meanwhile, all 117 patients who underwent surgery for distal hypospadias underwent surgery as a day case (no admission was required) (Table 1).

**Table 1:** Demographics and operative parameters of the post-onlay proximal hypospadias and distal hypospadias repair.

Variable	Proximal hypospadias post-onlay repair (n = 35)	Distal hypospadias (n = 117)
Mean age in years (range)	3.05 (1.1–10)	1.45 (0.5–4)
Mean operative time in minutes (range)	77.97 (60–110)	61.92 (35–105)
Mean duration of urinary catheterisation in days (range)	3 (3–3)	2.22 (1–3)
Mean follow-up duration in months (range)	10.94 (5–12)	9.63 (6–12)

**Figure 1:**
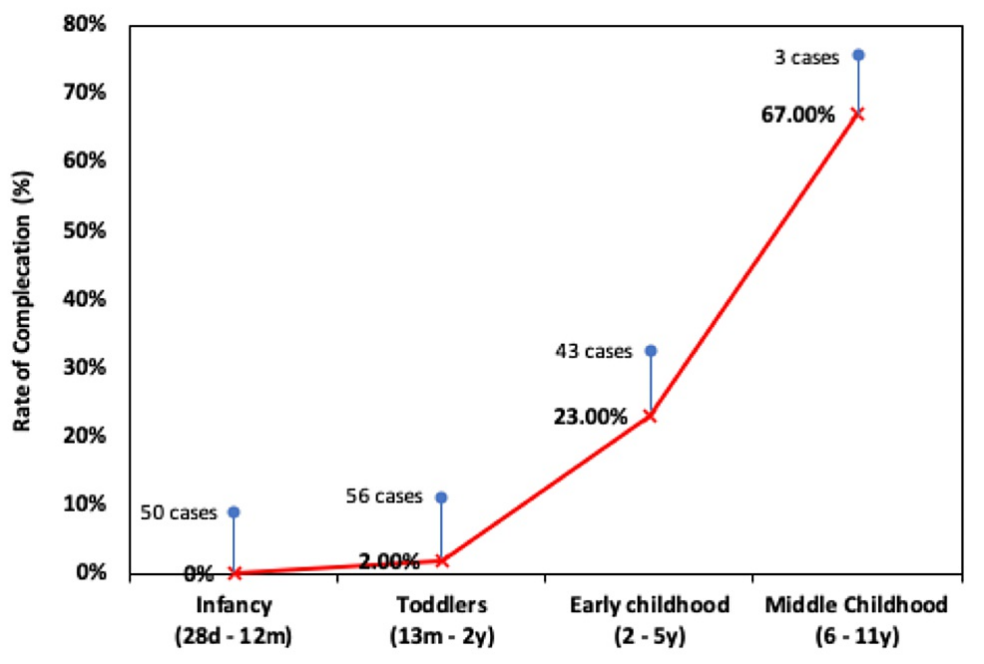
Rate of surgery complications against four different age groups. The percentages shown are with respect to the total number of cases in each group.

Figure 1 shows the rate of complications according to the age group of the study cases. While the rate of complication was 0% in the infant group, the complication rate increased with the age of the child. Further, while there was a relationship between the age and the rate of compilations, most of the older age cases developed only minor complications. With a particular focus on the two aforementioned main groups examined in this study (proximal post-onlay and distal hypospadias repair), it can be observed that four of 35 patients (11.4%) experienced complications in the post-onlay group. Of these, there were two (5.7%) cases of meatal stenosis, two (5.7%) cases of wound dehiscence, and no cases of diverticula or urethrocutaneous fistulas. Meanwhile, in the distal hypospadias group, one of 117 patients (0.9%) experienced a complication; the complication was meatal stenosis in a patient with distal penile hypospadias. However, there were no cases of wound dehiscence, UCFs, or diverticula. Overall, there was a significant correlation between whether the hypospadias was distal or proximal (following the onlay repair) and the occurrence of complications (p = 0.002). Furthermore, there was a significant correlation between the age of the patients and the complication rate (p = 0.06). The operative time was also found to be a significant factor influencing the occurrence of complications (p < 0.001). However, there was no significant correlation between the duration of urinary catheterisation and complications (p = 0.286) (Table 2).

**Table 2:** Complications in post-onlay proximal hypospadias and distal hypospadias repair.

Complication rates and types	Proximal hypospadias post-onlay repair (n = 35)	Distal hypospadias (n = 117)
Complication rate, n (%)	4 (11.4)	1 (0.9)
Meatal stenosis, n (%)	2 (5.7)	1 (0.9)
Wound dehiscence, n (%)	2 (5.7)	0 (0)
Urethrocutaneous fistula, n (%)	0 (0)	0 (0)
Diverticula, n (%)	0 (0)	0 (0)

The follow-up duration ranged between five months and 12 months. All patients were doing well at their final follow-up appointment in terms of the location of the meatus, urinary stream, and cosmesis. Specifically, the outcome of the procedure was evaluated both by clinical/physical examination and by asking the parents about the child’s urine stream following the procedure.

## Discussion

The anomalies associated with hypospadias are the ventral ectopic positioning of the urethral meatus, a penile curvature (chordee), and deficient ventral skin [12]. The position of the urethral meatus in hypospadias can occur between the perineum to the penile glans with the corpus spongiosum dividing more proximally in more severe forms [12,13].

One of the options available for the repair of proximal hypospadias is the traditional repair, which involves the formation of a neourethra. The urethral mobilisation and glanuloplasty post-onlay repair are emphasised in this article as they provide the least associated complication rates (especially fistulas and strictures) [14-20]. The components of the first part of the repair are correcting the penile curvature, excising the urethral plate, and harvesting a graft or flap that will be subsequently used for the neourethral plate creation. During the second part, the graft or flap is tubularised to reposition the urethral meatus to the targeted position in the glans [5,21]. The grafts most frequently used are from preputial skin and buccal mucosa. Meanwhile, an alternative to grafts is the use of Byars flaps (from the dorsal prepuce) [22]. The main hallmark of the repair is the creation of the neourethral plate and tubularisation to create a neourethra [14]. On the other hand, several repair techniques are available to repair distal hypospadias. These options include meatal advancement and glanuloplasty, tubularisation, and flap techniques [6]. One of the most commonly used techniques used to repair distal hypospadias is the meatal advancement and glanuloplasty technique due to the lower complication rates associated and good cosmetic outcomes [6].

In this study, a single-stage surgical approach was used, whereby the first part involved the repair of proximal hypospadias after an onlay procedure (where the meatus was corrected with urethral mobilisation and glanuloplasty) was combined with a modified second part of the repair, which is usually used to repair distal hypospadias, i.e., the meatal advancement and glanuloplasty technique. This technique was predicted to provide satisfactory outcomes and decrease complication rates. It also provides the advantage of shorter operating times, day-case for urethral mobilisation and glanuloplasty (after onlay repair), and shorter durations of urinary catheterisation. In comparison to other studies, this study found that urethral mobilisation and glanuloplasty after onlay repair for proximal hypospadias resulted in a complication rate of 11.4%, which is lower than the rate described in a systematic review of eight studies where the complication rate was 24.2% using the TIP procedure to repair proximal hypospadias [23]. Furthermore, there were no occurrences of UCFs following this technique (post-onlay repair) in this study, whereas Castagnetti et al. found a 24.2% occurrence of fistulas and dehiscence post-TIP repairs of proximal hypospadias [23]. The pre-existing hypotheses behind the high rates of fistulas and dehiscence using the traditional two-stage approach are the high urethral pressures caused by the long suture lines used to create the neourethra and inadequate support within the newly constructed urethra [10,11]. Therefore, it was predicted that the absence of postoperative UCFs in this study is a result of using glanuloplasty rather than neourethra formation with this technique following the initial onlay repair of hypospadias. Similarly, in two other studies, modifications were done to decrease complication rates, especially fistulas and dehiscence, by strengthening the repair with additional layers such as tunica vaginalis coverage, both of which rendered reduced complication rates, especially fistulas [10,24].

Furthermore, in this study, the operative time of this technique in the post-onlay group for proximal hypospadias repair was only 78 minutes. The urinary catheter remained in situ for a mean of three days postoperatively. Furthermore, the duration of hospitalisation for all patients was also only one day. Meanwhile, in other studies, following the traditional hypospadias repair or distal hypospadias, the urinary catheter remained in situ for 10-14 days and the duration of antibiotic administration was approximately 10 days [25].

Finally, the technique (post-onlay repair) used in this study yielded good outcomes in terms of the postoperative appearance of the penis, the meatal location, and the patients’ or their parents’ subjective views of the urinary steam. However, no objective measures of urinary functions, such as uroflow, were used.

The study found that in the distal hypospadias group, only one patient experienced a complication (0.9%), meatal stenosis. There were no incidences of wound dehiscence, UCFs, or diverticula. This compares to other studies which showed minimal complication rates associated with urethral advancement and glanuloplasty in the repair of distal hypospadias [26-28]. This is likely due to a variety of factors including the fact that the procedure is traditionally done in distal hypospadias cases. Moreover, the lower complication rate might be linked to the virginity of the tissue as well as the location of the meatus being at the glans penis, which requires less mobilisation. Furthermore, a significant correlation was found between the severity of the hypospadias and the complication rate (p = 0.002). The operative time was also found to be a significant factor influencing the occurrence of complications (p < 0.001).

There was a large difference between the post-onlay proximal hypospadias repair group size and the distal hypospadias group size, which limits the use of comparing them (35 post-onlay proximal hypospadias patients and 117 distal hypospadias patients). However, four of 35 patients experienced complications in the post-onlay repair group (11%) and one of 117 patients experienced a complication from the distal hypospadias cases repaired (0.9%). Furthermore, there were two postoperative cases of wound dehiscence in the post-onlay group (5.7%) and two cases of meatal stenosis (5.7%). Meanwhile, there was only one case of meatal stenosis in the postoperative distal hypospadias group (0.9%).

Several limitations can be identified in this study. First, the duration of follow-up was limited and did not allow for the long-term follow-up of patient outcomes in terms of sexual and urinary function as well as delayed complications. Second, there were no measures of objective functional outcomes used. Moreover, this was a single-centre study with a limited number of patients. This study was also limited by the lack of comparison of this technique (post-onlay repair of proximal hypospadias) to the traditional repair of proximal hypospadias. Furthermore, other limitations were the limitations in the group that were suitable for this procedure as cases complicated by fistulas or tethering would not be fit to undergo this approach.

## Conclusions

The urethral mobilisation and glanuloplasty technique for the post-onlay repair of proximal hypospadias resulted in short operative times, a short duration of urinary catheterisation, and a short hospitalisation duration. Moreover, although the traditional repair has proven to result in lower complication rates, particularly of fistulas and dehiscence, the modification to this technique (following onlay repair) in this study, which uses the method normally used to repair distal hypospadias, resulted in even lower complication rates than those reported in the literature. Furthermore, there remains a need for well-designed studies to compare this technique (which involves urethral mobilisation and glanuloplasty following onlay repair) to the traditional two-stage approach to proximal hypospadias. Furthermore, there is a need to conduct studies with longer follow-up durations and objective measures of function to provide a better comparison between the different techniques used.
